# Aquatic Macrophytes in the Remediation of Atrazine in Water: A Study on Herbicide Tolerance and Degradation Using *Eichhornia crassipes*, *Pistia stratiotes*, and *Salvinia minima*

**DOI:** 10.1021/acsomega.4c10903

**Published:** 2025-03-15

**Authors:** María Carolina Ramírez Hernandez, Jesley Nogueira Bandeira, Deisy Alexandra Rosero Alpala, Lucrecia Pacheco Batista, Mayara Alana Silvestre Araújo, Paulo Sergio Fernandes das Chagas, Daniel Valadao Silva, Elis Regina Costa de Morais

**Affiliations:** † Department of Agronomic and Forest Sciences, Federal University of the Semiarid-UFERSA, AV. Francisco Mota, 572 - Pres. Costa E Silva, RN, Mossoró, 59625-900 Rio Grande do Norte, Brazil; ‡ Engineering Center, Federal University of the Semiarid-UFERSA, AV. Francisco Mota, 572 - Pres. Costa E Silva, RN, Mossoró, 59625-900 Rio Grande do Norte, Brazil

## Abstract

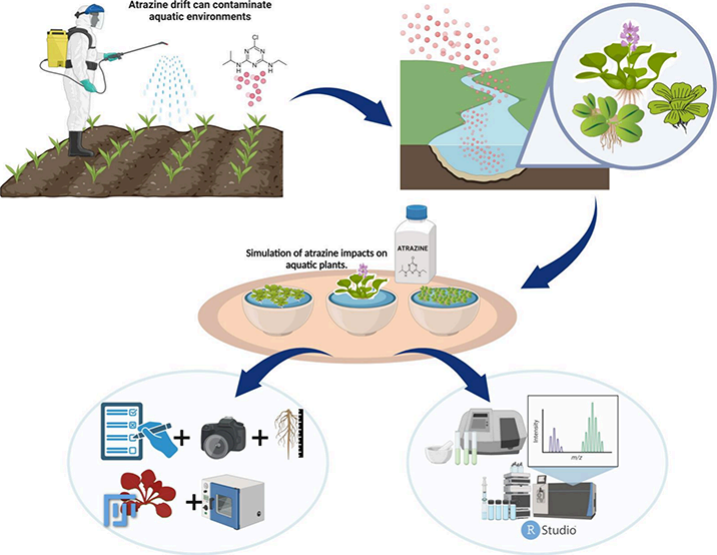

Aquatic macrophytes can be used for herbicide remediation provided they exhibit tolerance to the contaminants. This research assessed the remediation potential of *Salvinia minima*, *Echhornia crassipes*, and *Pistia stratiotes*, some common aquatic macrophytes native to Brazil, and their tolerance to atrazine, an herbicide commonly detected in waterbodies. Plants were cultivated under controlled conditions with five atrazine concentrations (0, 2, 20, 200, and 1000 μg L^–1^) for 15 days. *S. minima* and *E. crassipes* tolerated atrazine concentrations equal to or less than 20 μg L^–1^ and died at 200 and 1000 μg L^–1^, indicating the herbicide’s potential toxicity and its selectivity against sensitive species. *P. stratiotes* tolerated the herbicide concentration up to 200 μg L^–1^ and had its growth reduced at 1000 μg L^–1^. All species demonstrated the ability to reduce atrazine concentrations in water at 20 μg L^–1^ or lower, *E. crassipes* being the most efficient, reducing concentrations by 43% and 22% at 2 and 20 μg L^–1^, respectively. Atrazine levels within Brazilian (2 μg L^–1^ by CONAMA 2005) and European (0.1 μg L^–1^ by Directive 2013/33) regulatory limits do not selectively affect these species. Thus, they show potential for use in arazine phytoremediation programs.

## 1 Introduction

Herbicides are among the main pollutants of aquatic environments, being frequently reported as contaminants in reservoirs of surface and groundwater^1−6^ These substances can cause potential harmful effects to nontarget organisms such as microorganisms, insects, algae, and fish, resulting in direct impacts on aquatic ecosystems.^7−11^ The herbicide’s ability to cause negative impacts on the environment varies according to the physicochemical characteristics of the molecule and its interaction with environmental components.^12^

Atrazine [6-chloro-N-ethyl-N′-isopropyl-1,3,5-triazine-2,4-diamine] is one of the most widely used herbicides for weed control in various crops, such as corn, sorghum, sugar cane, and pineapple.^13^ Its molecules have the highest number of records as groundwater contaminants around the world,^14^ possibly due to its excessive use associated with its high persistence characteristic,^15^ which makes atrazine one of the main herbicides of environmental relevance.

An effective strategy for reducing the impacts generated by the accumulation of herbicides in an aquatic environment is bioremediation.^16,17^ Bioremediation encompasses biochemical processes involving the use of plants and microorganisms, resulting in both *in situ* and *ex situ* reduction of concentrations of contaminants, both organic and inorganic.^18^ The use of plants to remediate aquatic environments can be an efficient and cost-effective alternative, since the use of tolerant species can contribute to reducing contaminant concentrations in water and mitigate their direct and indirect impacts on the environment. Some studies have shown that aquatic macrophytes such as Water Hyacinth (*Eicchhornia crassipes* Mart.) Solms 1883), Water Spangles (*Salvinia minima* Baker, 1886), and Water Lettuce (*Pistia stratiotes* Kodda-Pail Adans. 1763) have been effective in removing herbicides such as Hexazinone and Clomazone from aquatic environments.^19−21^

Contaminant tolerance is an essential factor for selecting an herbicide-remediating species. This tolerance should be analyzed for concentrations within the expected limits set by the legislation of each locality, but it also needs to be evaluated for critical levels. For example, in Brazil, the permitted limit of atrazine in drinking water is 2.0 μg L^–1^,^22^ while in the European Union, this value is 0.1 μg L^–1^.^23^ Despite these established regulations, concentrations exceeding the maximum allowed limit for this herbicide have been found in aquatic environments.^24−26^ In the state of Ceará, the most significant concentration of atrazine was observed reaching 7 μg L^–1^, a value that exceeds the permitted limits for freshwater in Brazil.^5^

High concentrations of herbicides in water can be plant-selecting agents, making phytoremediation unfeasible under some conditions. In this context, this research had the following objectives: (I) to determine the maximum tolerance of three aquatic macrophytes *E. crassipes*, *S. minima*, and *P. stratiotes* to atrazine, and (II) to evaluate the efficiency of herbicide remediation of these three aquatic macrophytes. This approach provides new insights into the potential of these species for phytoremediation under conditions where herbicide concentrations may exceed regulatory limits, contributing to more effective bioremediation strategies in aquatic environments.

## 2 Material and Methods

### 2.1 Collection and Preparation of Aquatic Macrophytes

The macrophytes Water Hyacinth (*Eichhornia crassipes*), Water Spangles (*Salvinia minima*), and Water Lettuce (*Pistia stratiotes*) were collected from lotic environments. The first species was obtained from the Olho d’gua Velho Dam, Rio do Carmo (5°27′42.22″ S and 37°18′10.18′′ W), while the remaining species were collected from the Apodi-Mossoró River (5°11′42.89′′ S and 37°20′24.94” W). The plants were collected between 6 and 8 AM, with young plants in good condition selected from June to July 2021.

After being collected, the plants were stored in plastic containers with rainwater and placed in a greenhouse at the Federal Rural University of the Semi-Arid Region (UFERSA), in the city of Mossoró, Rio Grande do Norte, Brazil. The plants were disinfected by immersion in a 1% sodium hypochlorite solution for three min, followed by rinsing with running water for 10 min.

The plants were selected, isolated, propagated and maintained in 10, 20, and 25 L plastic containers with Hoagland’s nutrient solution^27^ for approximately one month under greenhouse conditions that ensured their adaptation.

### 2.2 Experimental Design

The treatments were arranged in a 3 × 5 factorial design, corresponding to three macrophytes (*Eichhornia crassipes*, *Pistia stratiotes*, and *Salvinia minima*) and five different doses of atrazine (0, 2, 20, 200, and 1000 μg L^–1^). The experimental units consisted of plastic containers with a volume of 10 L. For the treatments with *Pistia stratiotes*, 30 plants were placed per container; for *Salvinia minima*, 60 plants per container; and for *Eichhornia crassipes*, one plant per container. This difference was due to variations in leaf and root sizes among the species and the average density of their occurrence in the natural habitat. The experiment lasted for 15 days, a period selected to avoid internal competition among the species that could reduce their growth.

### 2.3 Herbicide Application

The herbicide application took place 1 h after transferring the plants to the 10 L plastic containers, using automatic pipettes. The water used was from a municipal supply, and the experiments were conducted in a closed greenhouse environment to prevent external contamination. After herbicide application, water samples were collected from the containers to ensure that the previously stipulated concentrations were maintained in each delineated treatment.

### 2.4 Evaluation of the Sensitivity of Macrophytes

#### 2.4.1 Intoxication

The visual signs of intoxication of the species were assessed 2, 4, 6, 8, 10, 12, and 15 days after herbicide application (DAA). The plants were assessed for toxicity symptoms that are typically associated with atrazine exposure. The rating scale used ranged from 0% to 100%: absence of intoxication (0%), mild intoxication (1% to 30%, mild chlorosis), moderate intoxication (31% to 69%, severe chlorosis, mild to moderate necrosis), severe intoxication (70% to 99%, severe chlorosis, and severe necrosis), and plant death (100%).

#### 2.4.2 Leaf Area

The leaf area (LA) was determined from photographic records taken at 1, 5, 9, 13, and 15 DAA, with the images digitally processed using the ImageJ software (S1) (National Institute of Health, NY, USA).

#### 2.4.3 Root Growth

To calculate the root growth index (RGI), photographic records were used from day 01 (T1) and day 15 after application (DAA) (T2), with the images digitally processed using the ImageJ software (National Institute of Health, NY, USA). The *RGI* was calculated using the equation below:
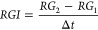
where *RG*_1_ and *RG*_2_ are the root growth of the plant at time 1 and time 2, respectively, and Δ*t* is the difference between time 1 and time 2 (days).^28,29^

#### 2.4.4 Total Dry Matter

At the end of the experiment (15 DAA), after determination of the LA and RGI, the plants were placed in paper bags and subjected to a forced-air oven at 70 °C until properly dried at a constant weight. After being dried, the material was weighed to determine the total dry matter (TDM).

#### 2.4.5 Chlorophyll Content

At the end of the experiment, the concentrations of photosynthetic pigments were determined and expressed in mg g^–1^. For this, chlorophylls a and b were extracted by macerating 200 mg of fresh mass, which were then transferred to small tubes containing 1 mL of 80% (v/v) acetone. Subsequently, the tubes were centrifuged and the supernatant was collected. This procedure was repeated four times, resulting in a total of 4 mL of supernatant containing chlorophyll. The supernatant was then used for absorbance readings in a spectrophotometer at wavelengths of 645, 649, and 665 nm for chlorophylls. Using these readings, the contents of chlorophyll *a*, *b*, and total were calculated, using the following equation:^30^
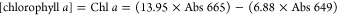

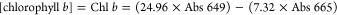

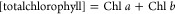


### 2.5 Evaluation of the Remediation Capacity of the Species

#### 2.5.1 Waste Atrazine in Water

Water samples were collected at specific intervals after herbicide application on days 2, 4, 6, 8, 10, 12, and 15. The water from each experimental unit was homogenized before collection to ensure sample uniformity. Subsequently, 1 mL samples were collected using syringes and filtered through Nylon membranes with a porosity of 0.22 μm. The filtered samples were stored in 1.5 mL vials and subsequently analyzed by ultrahigh-performance liquid chromatography (UHPLC).

#### 2.5.2 Chromatographic and Mass Spectrometry Conditions

The quantification of atrazine was performed on a UHPLC instrument connected to a Mass Spectrometer (LC-MS/MS). The UHPLC Nexera X2 (Shimadzu, Tokyo, Japan) is equipped with two LC-30AD pumps, a DGU-20A5R degasser, a Sil-30AC autosampler, a CTO-30AC column oven, and a CBM-20A controller. Separation occurred on a Restek column (Pinnacle DB AQ C18, 50 mm × 2.1 mm, 1.9 μm particles). The flow rate was 0.15 mL min^–1^ with an isocratic elution containing 64% of pump B, injection volume of 5 μL, and sampler, column oven temperatures set at 15 and 40 °C, respectively. Mobile phase A consisted of ultrapure water with 0.1% formic acid, while mobile phase B consisted of HPLC-grade acetonitrile.

The triple quadrupole mass spectrometer LCMS-8040 series (Shimadzu, Tokyo, Japan) with an electrospray ionization (ESI) source was operated in positive ionization mode. The parameters for multiple reaction monitoring (MRM) are summarized in Table 1. The interface voltage was set to 4.5 kV, the desolvation line temperature was 250 °C, the nebulizing nitrogen gas flow rate was 3 L min^–1^, the block temperature was 400 °C, the drying nitrogen gas flow rate was 15 L min^–1^, and the collision argon gas pressure was 230 kPa.

**Table 1:** Optimized Chromatographic Parameters^a^

		Quantification			Confirmation		
Herbicide	Retention time (min.)	MRM transition *m*/*z*	DP (V)	EC (V)	MRM transition *m*/*z*	DP (V)	EC (V)
Atrazine	1.910	216.10 > 174.10	–18	–16	216.10 > 96.15	–18	–25

a Multiple reaction monitoring (MRM), declustering potential (DP), collision energy (CE), mass-to-charge ratio (*m*/*z*), and voltage (V).

### 2.6 Statistical Analyses

Statistical analysis was conducted using RStudio software, version 2023.06.1+524. To assess the homogeneity of variances, the Levene test was applied, while the normality of errors was checked using the Shapiro-Wilk test. A significance level of *p* < 0.05 was considered for both analyses. When the *p*-value of the Levene test was greater than 0.05, indicating homoscedasticity of variances, and the *p*-value of the Shapiro-Wilk test was also greater than 0.05, indicating normality of errors, and the data were considered suitable for parametric analysis (ANOVA). For comparisons between means, the Tukey test was used, with a significance level of *p* < 0.05.

## 3 Results and Discussion

### 3.1 Intoxication

**Figure 1:**
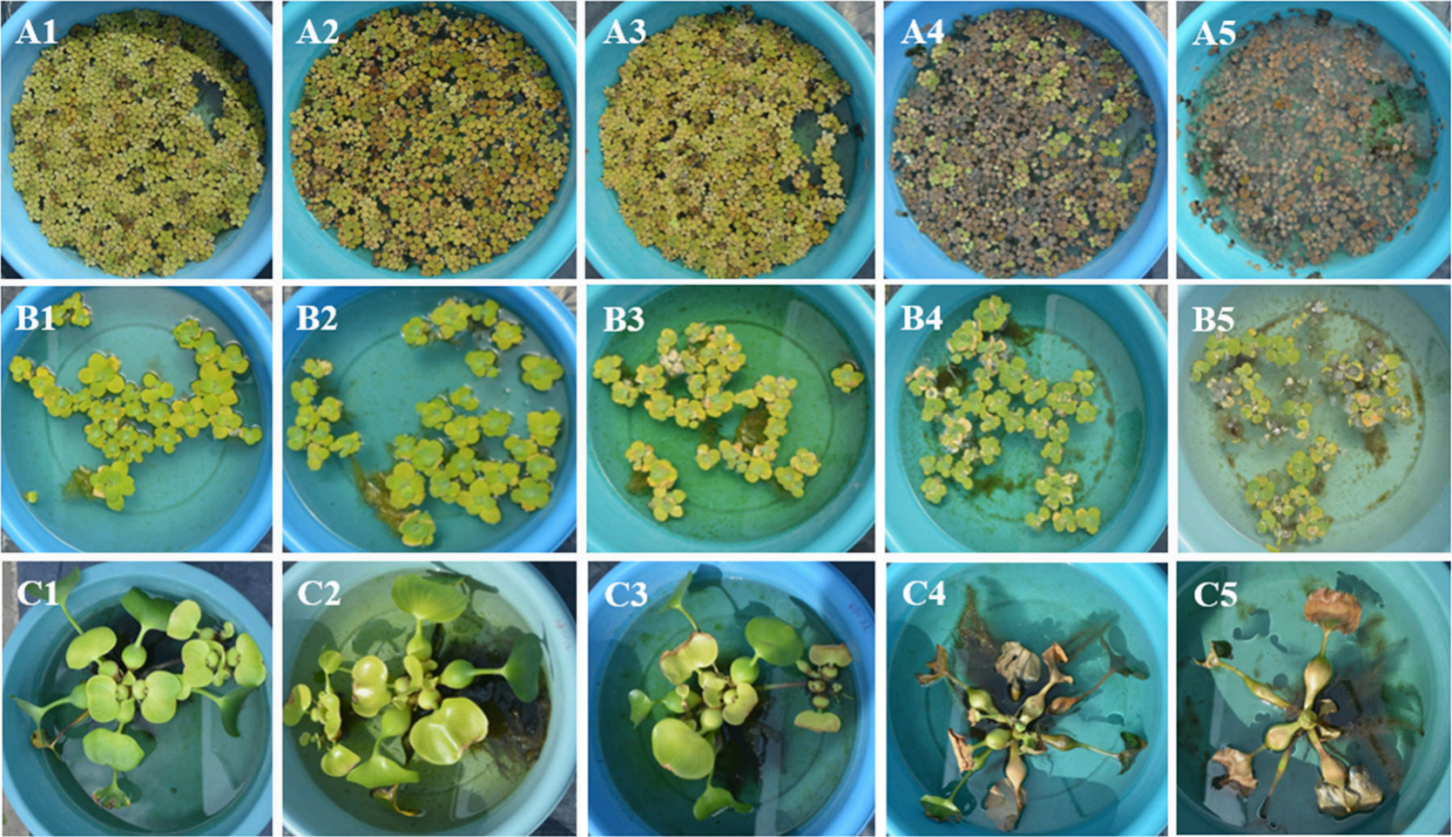
Visual signs of intoxication of *S. minima* (A), *P. stratiotes* (B), and *E. crassipes* (C) plants at concentrations 0 (1), 2 (2), 20 (3), 200 (4), and 1000 (5) μg L^–1^ of atrazine, at 15 days after application (DAA).

All aquatic macrophyte species used in the study showed symptoms of intoxication when exposed to doses of the herbicide atrazine (Figure 1). The visual symptoms were characterized by chlorosis at the leaf margins progressing toward the center, followed by leaf necrosis and plant death. These symptoms are common in species susceptible to herbicides that inhibit photosystem II.^31^

**Figure 2:**
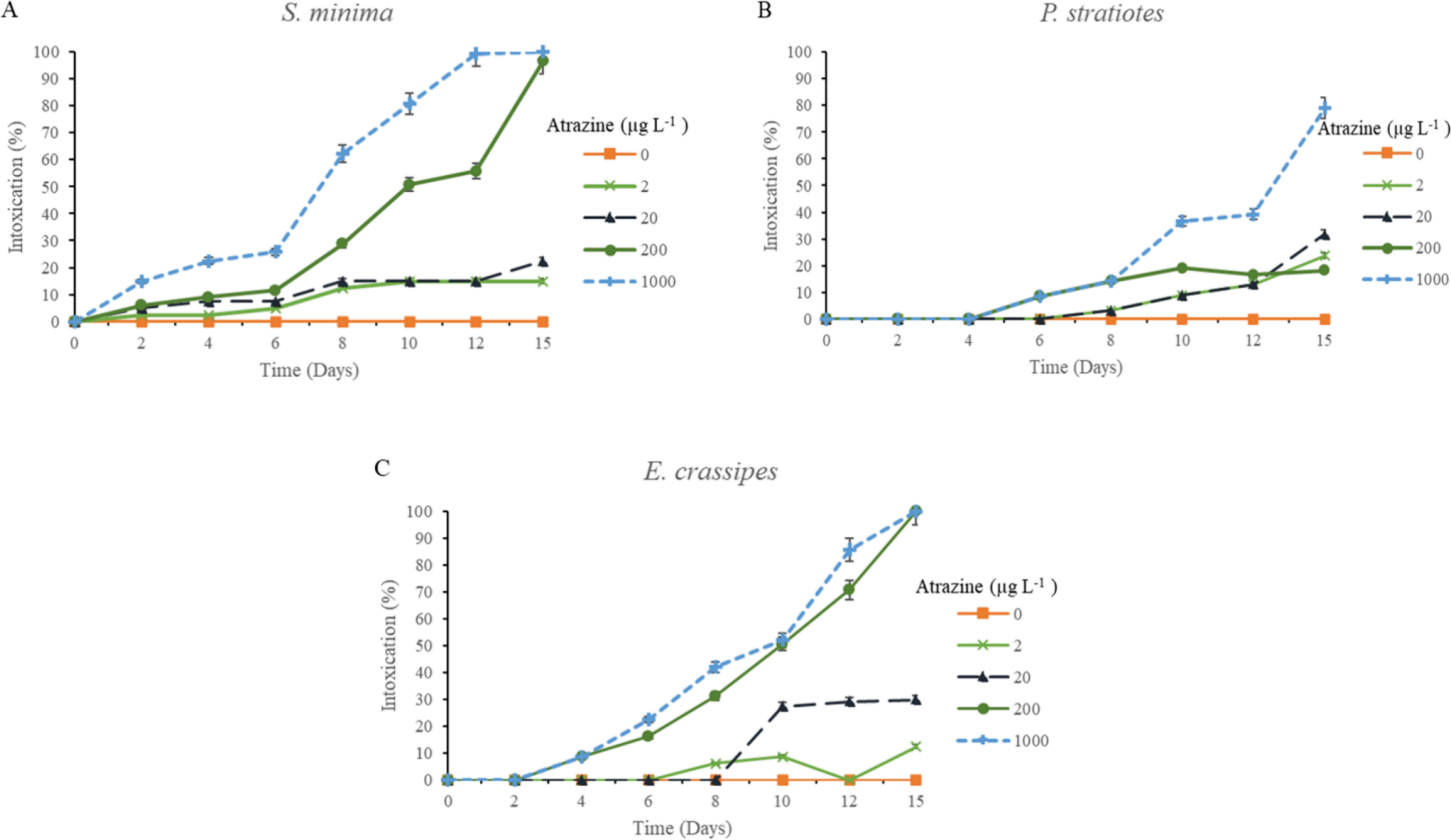
Visual signs of intoxication levels (%) for *S. minima* (A), *P. stratiotes* (B), and *E. crassipes* (C) at concentrations of 0, 2, 20, 200, and 1000 μg L^–1^ atrazine as a function of days after application (DAA). Error bars represent the 95% confidence intervals of the mean (*p* ≤ 0.05), as determined by Tukey’s test.

The species *S. minima* exhibited significant levels of intoxication when exposed to increasing concentrations of atrazine. Intoxication rates of 96% (severe necrosis) and 100% (plant death) were observed when the patients were exposed to concentrations of 200 and 1000 μg L^–1^ of atrazine, respectively. In contrast, at concentrations of 2 and 20 μg L^–1^, the intoxication rates were considerably reduced, registering only 12% and 22%, respectively (Figure 2).

*E. crassipes* demonstrated sensitivity to the herbicide, with a level of intoxication of 100% (plant death) at concentrations of 200 and 1000 μg L^–1^. Conversely, at concentrations of 2 and 20 μg L^–1^, low toxicity was observed, registering only 12% and 30%, respectively (Figure 2). Correa dos Santos et al.,^32^ studying the species *E. crassipes* and *P. stratiotes*, exposed to environments contaminated with atrazine, found that *E. crassipes* exhibited maximum sensitivity, resulting in species mortality at a concentration of 1000 μg L^–1^. These results corroborate ours once this species also demonstrated sensitivity to concentrations above 200 μg L^–1^.

On the other hand, the species *P. stratiotes* demonstrated greater tolerance to the herbicide compared to the other species studied. At concentrations of 200 and 1000 μg L^–1^, intoxication levels of 18% and 79% (severe chlorosis) were observed. When exposed to concentrations of 2 and 20 μg L^–1^ atrazine, they exhibited intoxication rates of 23% and 31%, respectively (Figure 2).

Some studies indicate that aquatic macrophytes may react sensitively to certain herbicides, while in other cases, the effect on the plant may be reduced or considered insignificant.^33−35^ Additionally, this sensitivity can fluctuate throughout the plant’s life cycle and in various stages of its growth.^32,36,37^

Atrazine induced phytotoxicity, manifesting as chlorosis and necrosis, ultimately resulting in the death of susceptible plants. The atrazine mechanism of action involves blocking the electron flow from photosystem II to photosystem I due to competition for the electron-binding site on the D1 protein. With this blockage, there is an accumulation of electrons that cause lipid peroxidation and membrane disintegration.^38−41^ The high levels of phytointoxication in aquatic macrophytes when subjected to concentrations higher than 200 μg L^–1^ may have been caused by the destruction of chloroplasts in the leaves and whitening due to photo-oxidative damage to chlorophylls, resulting from the absorption of the herbicide by the plants.^42,43^

However, some plant species, such as *P. stratiotes*, exhibit remarkable tolerance to environments contaminated with atrazine. This capacity is attributed to its ability to generate new rosettes or rooted branches. These structures promote an interconnected communication that when not fragmented allows a more effective adaptation to the environment. Furthermore, this interconnection facilitates a better utilization of the available resources for plant development.^36^ This phenomenon suggests the existence of mechanisms by which the plant can mitigate the toxic effects of atrazine. Thus, it is presumed that species such as *S. minima* and *E. crassipes* lack mechanisms to tolerate and minimize the effects of atrazine at doses of 200 and 1000 μg L^–1^.

### 3.2 Plant Growth

#### 3.2.1 Leaf Area

**Figure 3:**
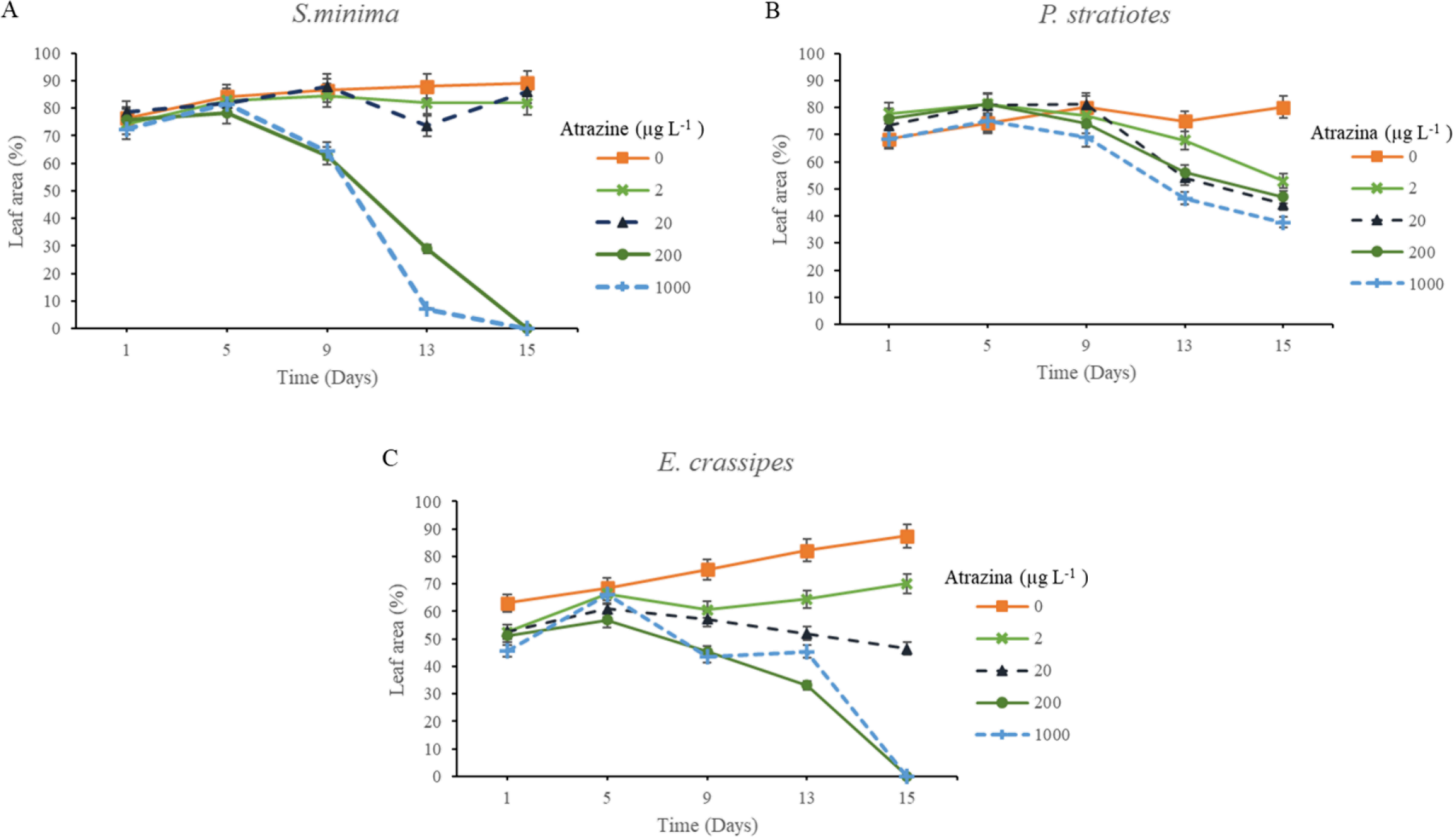
Leaf area growth (cm^2^) for *S. minima* (A), *P. stratiotes* (B), and *E. crassipes* (C) at concentrations of 0, 2, 20, 200, and 1000 μg L^–1^ of atrazine over days after application (DAA). Error bars represent the 95% confidence intervals of the mean (*p* ≤ 0.05), as determined by Tukey’s test.

Atrazine induced a progressive reduction in the leaf area of *S*. *minima* and *E. crassipes* species when exposed to doses of 200 and 1000 μg L^–1^ (Figures 3A and 3C), observed from the ninth day for *S. minima* and from the 13th day for *E. crassipes*. This decrease was gradual over time, resulting in the total death of the plants by the 15th day. On the other hand, at concentrations of 2 and 20 μg L^–1^, no significant variation in leaf area was observed for *S. minima* and *E. Crassipes* species (Figures 3A and 3C).

Herbicides that inhibit photosystem II, such as atrazine, isoproturon, diuron, and simazine, of the urea/triazine type, have been found to affect the growth of plants such as *E. canadenses*, *M. spicatum*, *Persicaria amphibia*, and *Glyceria maxima* at concentrations higher than 75 μg L^–1^. This effect may be related to the energy expended by the plant to activate its antioxidant system and stabilize oxidative effects.^44,45^

During the first 9 DAA of the herbicide, a tendency to maintain leaf area was observed in plants belonging to the *P. Stratiotes* species (Figure 3B). This growth pattern has been observed in various plant species during the early stages of herbicide exposure.^21,46^

The reduction observed in the leaf area for all species at doses of 200 and 1000 μg L^–1^ likely occurred because of the inhibition of plant growth by the herbicide, resulting in the interruption of photosynthesis. This phenomenon occurs due to the blocking of electron flow between photosystems II and I, which prevents the production of adenosine triphosphate (ATP) and nicotinamide adenine dinucleotide phosphate (NADPH), as well as the subsequent fixation of CO_2_. These processes are essential for biomass accumulation in plants.^21,47^

#### 3.2.2 Dry Matter

**Figure 4:**
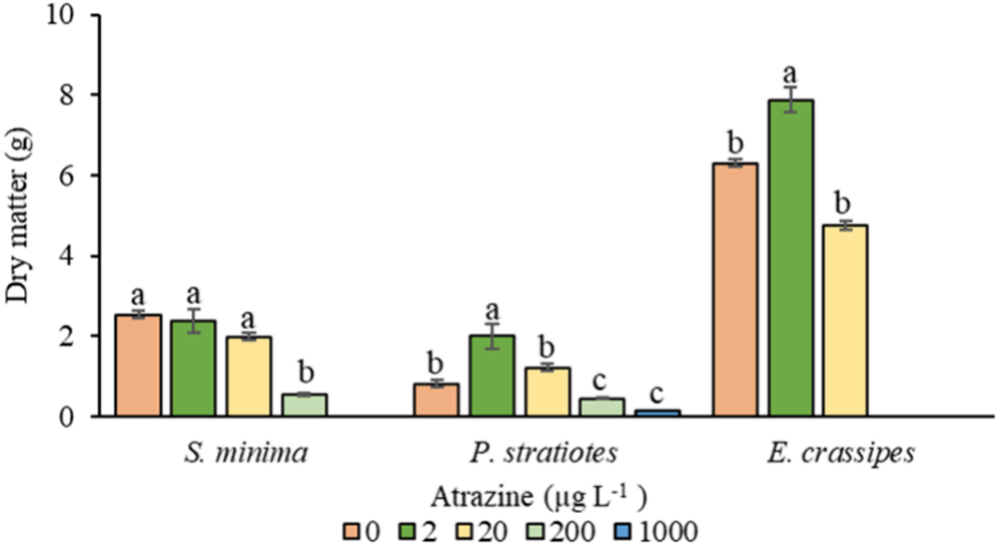
Dry matter of *S. minima*, *P. stratiotes*, and *E. crassipes* at concentrations of 0, 2, 20, 200, and 1000 μg L^–1^ of atrazine. Different letters indicate significant statistical differences by the Tukey test (*p* ≤ 0.05). The vertical bars indicate the standard error of the mean (*n* = 4).

Regarding dry matter, there were increases of 14% and 29% for *S. minima* at concentrations of 2 and 20 μg L^–1^, respectively, compared to the control. However, at concentrations of 200 and 1000 μg L^–1^, dry matter was reduced by 77% and 100%, respectively (Figure 4). For *E. crassipes*, an increase of 26% and 34% in dry matter was observed, respectively, when exposed to concentrations of 2 and 20 μg L^–1^. The highest doses (200 and 1000 μg L^–1^) resulted in plant death (Figure 4), which may be associated with direct damage to plant cells and tissues. This damage possibly caused severe disruption of photosynthetic activity, oxidative stress, and metabolic failure. These processes may have contributed to necrosis, reduced growth, and ultimately the death of the exposed plants.^48−51^

The dry matter of *P. stratiotes* increased by 40 and 32% at doses of 2 and 20 μg L^–1^, respectively, compared to the control. However, at concentrations of 200 and 1000 μg L^–1^, there were reductions of 45% and 89%, respectively (Figure 4). These results suggest that macrophytes may develop adaptation mechanisms to herbicide exposure, such as the activation of specific metabolic pathways that promote growth and biomass production under stressful conditions.^50,52,53^

#### 3.2.3 Root Growth

**Figure 5:**
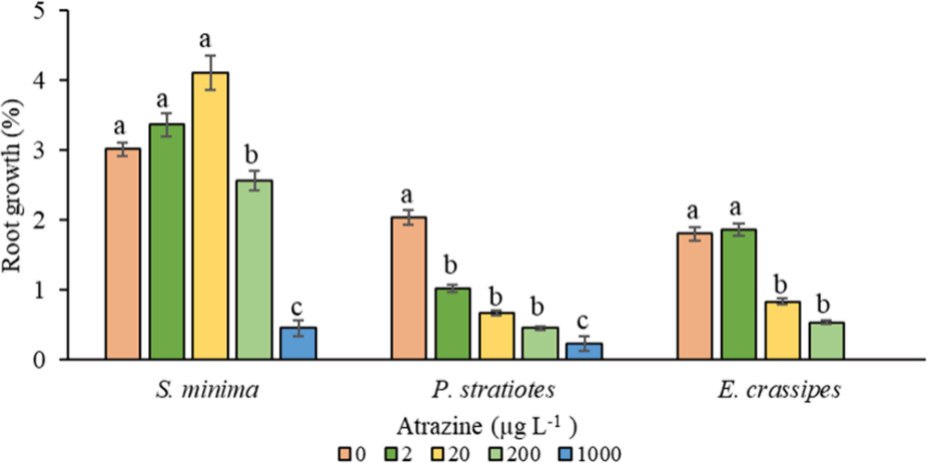
Root growth index (%) for *S. minima*, *P. stratiotes*, and *E. crassipes* at atrazine concentrations of 0, 2, 20, 200, and 1000 μg L^–1^ as a function of days after application (DAA). Different letters indicate significant statistical differences by the Tukey test (*p* ≤ 0.05). The vertical bars indicate the standard error of the mean (*n* = 4).

The root growth index (RGI) of *S. minima* increased by 11.5% and 46% at doses of 2 and 20 μg L^–1^, respectively (Figure 5). However, at a concentration of 200 μg L^–1^, a 13% reduction in RGI was observed, while at a dose of 1000 μg L^–1^, this reduction was 78%. This behavior suggests that macrophytes may exhibit increased root development as a compensatory strategy in response to herbicide-induced damage to the shoot. The expansion of the root system can play a crucial role in stabilizing the affected plant and providing supplemental support for subsequent growth.^21,36^ As a comparison, in wheat species dominated by genetically similar strains of *A. ureafaciens* (Kytasatospora), an increase in root growth in response to atrazine-induced stress has been observed, which enhances competitiveness in the rhizosphere.^54^

*P. stratiotes* and *E. crassipes* species did not show a significant increase in RGI with an increasing dose (Figure 5). For *P. stratiotes*, a decrease in RGI of 50%, 67%, 77%, and 90% was observed at concentrations of 2, 20, 200, and 1000 μg L^–1^, respectively. Conversely, for *E. crassipes*, an increase of 3% in the RGI was observed at a concentration of 2 μg L^–1^. However, at concentrations of 20, 200, and 1000 μg L^–1^, there were reductions of 53%, 70%, and 100%, respectively. These results suggest that the adverse effects of atrazine-induced stress on root growth may be associated with the reduction of enzymatic activity induced by the herbicide.^51^

### 3.3 Biochemical Response of Plants to Atrazine

#### 3.3.1 Content of Chlorophyll *a* and *b* and Total Chlorophyll

**Figure 6:**
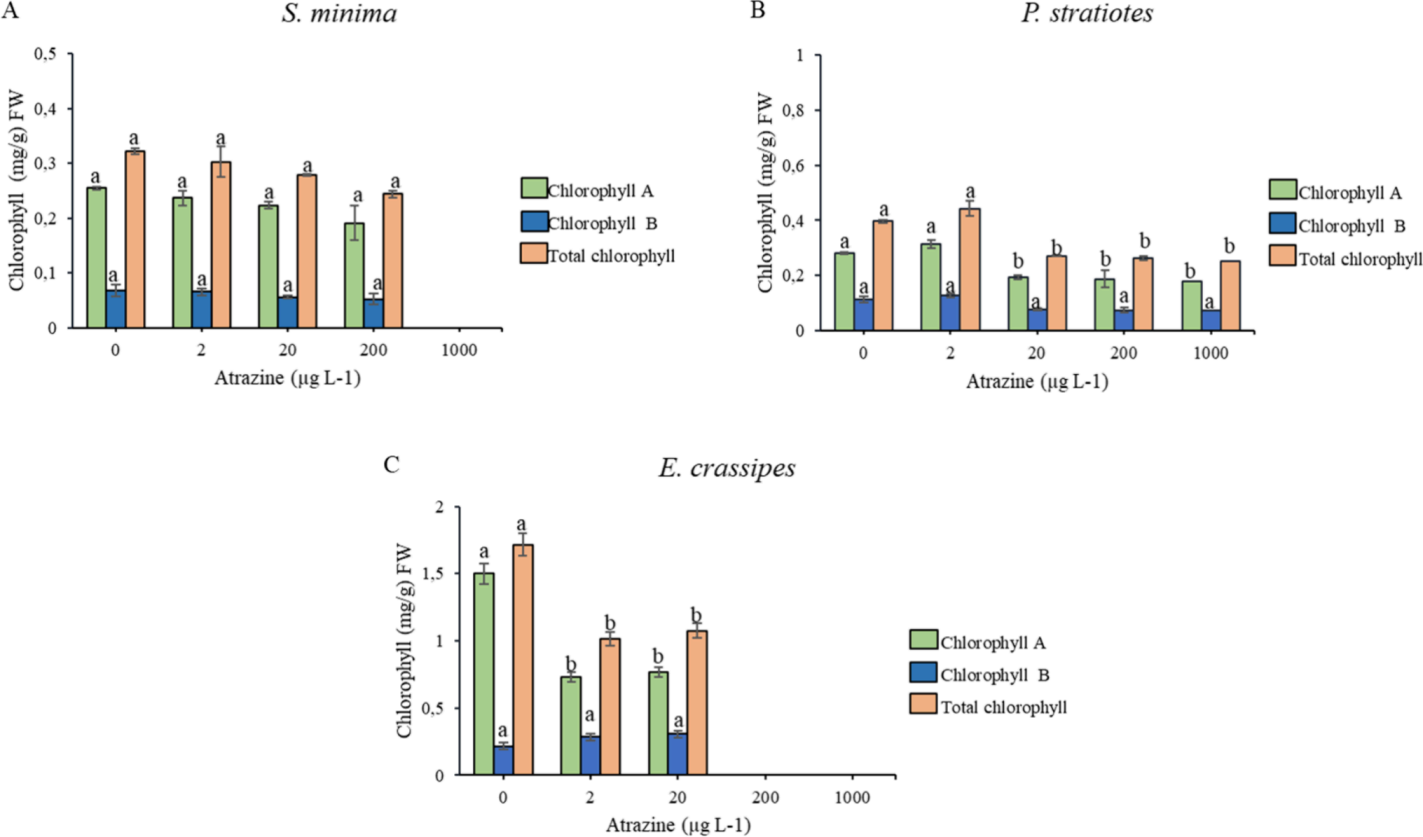
Chlorophyll *a* and *b* and total chlorophyll content in *S. minima* (A), *P. stratiotes* (B), and *E. crassipes* (C) at atrazine concentrations of 0, 2, 20, 200, and 1000 μg L^–1^, 15 days after application. Different letters indicate significant statistical differences by the Tukey test (*p* ≤ 0.05). The vertical bars indicate the standard error of the mean (*n* = 4).

The amount of chlorophyll *a*, chlorophyll *b*, and total chlorophyll in *S. minima* was not altered in response to any of the atrazine concentrations (Figure 6A). However, for *E. crassipes*, it was possible to observe a decrease in the content of chlorophyll *a* and total chlorophyll of 51% and 40% at the concentration of 2 μg L^–1^ of atrazine, while at the concentration of 20 μg L^–1^ there were reductions of 46% and 37% compared to the control (Figure 6C). For *P. stratiotes*, reductions of 31% in both chlorophyll *a* and total chlorophyll content were observed in plants exposed to concentrations of 20 μg L^–1^, 33% and 34% at atrazine concentration of 200 μg L^–1^, and 4% and 4.07% at a concentration of 1000 μg L^–1^. No significant differences in the chlorophyll *b* content were observed for these species (Figure 6B).

The stable chlorophyll concentrations observed in *S. minima* may suggest limited dissipation of photosynthetic energy within these plants.^55,56^ However, this observation differs from other studies as this species was identified as the most sensitive in this study, and it was expected to show lower chlorophyll levels. Generally, chlorophyll levels are considered reliable biomarkers of toxicity due to the photochemical reactions sensitive to PSII, which respond differently to the effects caused by pollutants.^56−58^

The stability of chlorophyll *a* and *b* and total chlorophyll in *P. stratiotes* at the lower herbicide doses (2 and 20 μg L^–1^) may be associated with the plant’s defense mechanisms under oxidative stress conditions. Under such conditions, it is possible that electron transport was not inhibited and that complete inactivation of the active reaction centers of PSII did not occur.^55,59^ Additionally, some findings also indicate an increase in chlorophyll in algae and aquatic plants exposed to low light levels or photosynthesis inhibition, especially due to PSII inhibition.^60,61^

### 3.4 Remediation Capacity of Macrophytes to the Atrazine

**Figure 7:**
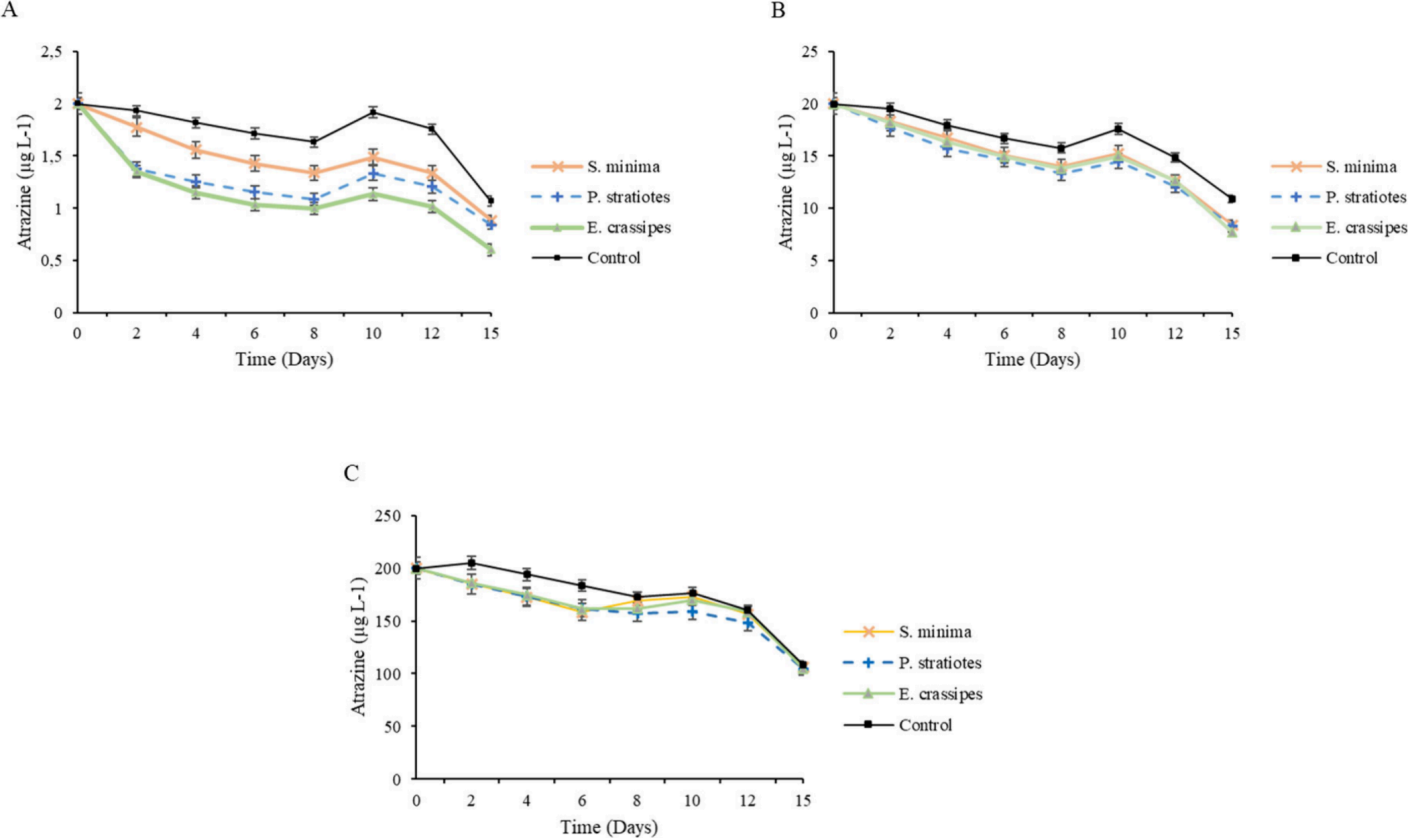
Remediation of atrazine by *S. minima*, *P. stratiotes*, and *E. crassipes* species at concentrations of 2 (A), 20 (B), and 200 μg L^–1^ (C), as a function of days after application (DAA). Error bars represent the 95% confidence intervals of the mean (*p* ≤ 0.05), as determined by Tukey’s test.

The macrophytes used in the study exhibited variations in their capacity to reduce atrazine levels when exposed to the contaminant (Figure 7A, 7B, and 7C).

When exposed to a concentration of 2 μg L^–1^ of atrazine, *S. minima, P. stratiotes*, and *E. crassipes* species were able to reduce the amount of the herbicide in the water by 17%, 21%, and 43%, respectively, at 15 DAA, compared to the control (Figure 7A). In addition, the control group also showed a reduction of 46% in herbicide concentration at 15 DAA. The reduction observed in the atrazine concentration in the control (without aquatic plants) may be attributed to the process of photodegradation induced by exposure to sunlight, resulting in the breakdown of atrazine molecules into smaller compounds.

This process is influenced by various factors, including sunlight intensity, temperature, and presence of microorganisms, among others.^6,51,62,63^ The absorption of atrazine by aquatic plants does not ensure its complete remediation, requiring other processes such as accumulation in biomass or degradation through the plant’s metabolism itself.^63,64^

When exposed to a concentration of 20 μg L^–1^, the three species used in the study showed similar behavior in decontaminating the aqueous medium with the herbicide. *E. crassipes*, *P. stratiotes*, and *S. minima* were able to reduce the concentration of atrazine by 22%, 23%, and 28% (15 DAA), respectively, compared to the control (Figure 7B).

At a concentration of 200 μg L^–1^, despite showing high sensitivity (Figure 2), *E. crassipes* and *S. minima* species were able to reduce the herbicide concentration by 14% and 13%, respectively, at 6 DAA (Figure 7C). From this day onward, these species exhibited increased sensitivity and mortality, resulting in an increase in herbicide concentration in the water until reaching levels similar to the control (15 DAA). Under these conditions, the species appeared to lose efficiency, possibly due to herbicide intoxication, which likely exceeded their tolerance capacity. On the other hand, *P. stratiotes* species decreased the herbicide concentration in the water by 12% compared to the control at 15 DAA (Figure 7C).

At a concentration of 1000 μg L^–1^, it was observed that the studied species were not able to remove atrazine from the aquatic environment due to the high sensitivity and intoxication promoted by exposure to this high concentration of herbicide.

Some studies demonstrate the efficiency of *S. minima* in the accumulation of heavy metals,^65−67^ highlighting the plant’s ability to absorb and retain harmful substances. Other researchers emphasize accumulation, in contrast to degradation, as one of the mechanisms involved in atrazine removal.^68,69^

*P. stratiotes* has been reported to be efficient in removing heavy metals from aquatic environments,^73^ and it is widely known as an excellent phytoremediator because of its rapid growth and root development in polluted environments.^70−74^ Research indicates that *P. stratiotes* can be used to remediate residues of clomazone, mesotrione, and saflufenacil in water during 25 days of exposure.^36,75^ These results demonstrate that this species can be an excellent alternative for remediating herbicides frequently found in aquatic environments.

*E. crassipes* species is capable of accumulating different pollutants in its roots and shoot.^76^ A study conducted by Correia et al.^77^ indicates that this macrophyte species can accumulate pollutants in its roots. Other studies also demonstrate the capacity of *E. crassipes* to remediate polluted waters and bioaccumulate heavy metals, pesticides, antibiotics, and organic matter.^62,78−81^ These outcomes highlight the different responses of aquatic macrophytes to herbicides, emphasizing the need to consider tolerance when assessing the impacts of herbicides on these ecosystems.

The transport of atrazine from agricultural areas to aquatic environments can elevate its concentration in water resources, thereby acting as a selective agent for aquatic plants. However, as demonstrated in this study, at lower concentrations (up to 20 μg L^–1^), plants naturally show remediation capacity.

Additional studies are essential to understand the remediation mechanisms of plants, once bioaccumulation may demand the removal of plant material from the water, or in case of rhizofiltration or phytodegradation, as observed in other species,^82−84^ decontamination can be an environmentally efficient technique.

## 4 Conclusions

The aquatic macrophytes *E. crassipes*, *S. minima*, and *P. stratiotes* demonstrated tolerance to atrazine concentrations of up to 20 μg L^–1^. For atrazine concentrations exceeding 200 μg L^–1^, the study indicates that the macrophytes may experience serious growth impairment or cessation, suggesting that the herbicide could act as a species selector in this environment.

The species *E. crassipes*, *S. molesta*, and *P. stratiotes* represent potential alternatives for the remediation of freshwater ecosystems impacted by the use of atrazine. It is important to emphasize the need for further studies to elucidate the remediation mechanisms of these species as well as to test the technique in natural environments and in a combined approach.

## Supporting Information Available

Method used to calculate leaf area in the treatments (PDF)
