# Supplementary material for: Aquatic Macrophytes in the Remediation of Atrazine in Water: A Study on Herbicide Tolerance and Degradation Using Eichhornia crassipes, Pistia stratiotes, and Salvinia minima
Source: ACS Omega. 2025 Mar 15;10(11):11264–73. doi: 10.1021/acsomega.4c10903 (PMC11948152; doi:10.1021/acsomega.4c10903)
Supplement: Supplementary file 1 — ao4c10903_si_001.pdf [file ao4c10903_si_001.pdf]

AQUATIC MACROPHYTES IN THE REMEDIATION OF ATRAZINE IN WATER: A STUDY ON HERBICIDE TOLERANCE AND DEGRADATION USING *Eichhornia crassipes, Pistia stratiotes* and *Salvinia minima*.

María Carolina Ramírez Hernandez[a*], Jesley Nogueira Bandeira [a], Deisy Alexandra Rosero Alpala[a], Lucrecia Pacheco Batista[a], Mayara Alana Silvestre Araújo[a], Paulo Sergio Fernandes das Chagas[a], Daniel Valadao Silva[a], Elis Regina Costa de Morais[b]

[a] Department of Agronomic and Forest Sciences, Federal University of the Semiarid-UFERSA, AV. Francisco Mota, 572 - Pres. Costa E Silva, RN, Mossoró, 59625-900, Rio Grande do Norte, Brazil,

[b]Engineering Center, Federal University of the Semiarid-UFERSA, AV. Francisco Mota, 572 - Pres. Costa E Silva, RN, Mossoró, 59625-900, Rio Grande do Norte, Brazil,

Email:maria.ramirez@alunos.ufersa.edu.br

Supporting Information

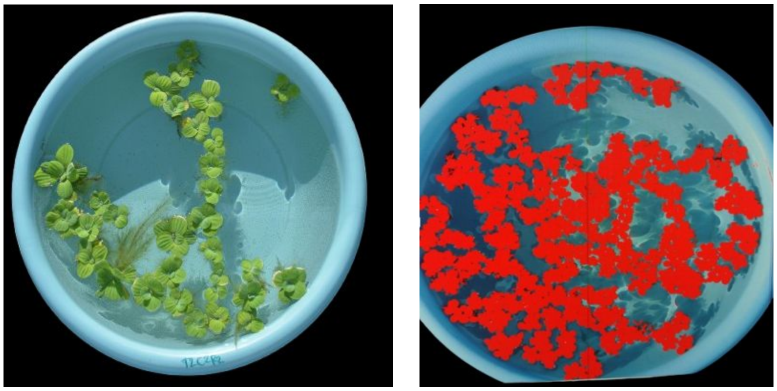

Figure S1. Example of the photographic method used to calculate leaf area in the treatments. The image on the left shows a representative photograph taken during the experiment, while the image on the right presents another photograph processed using ImageJ software to isolate and quantify the leaf area (highlighted in red). This procedure was applied to assess the effect of treatments on plant growth.
